# Self-sensing, tunable monolayer MoS_2_ nanoelectromechanical resonators

**DOI:** 10.1038/s41467-019-12795-1

**Published:** 2019-10-23

**Authors:** Sajedeh Manzeli, Dumitru Dumcenco, Guilherme Migliato Marega, Andras Kis

**Affiliations:** 1 Electrical Engineering Institute, École Polytechnique Fédérale de Lausanne (EPFL), 1015 Lausanne, Switzerland; 2 Institute of Materials Science and Engineering, École Polytechnique Fédérale de Lausanne (EPFL), 1015 Lausanne, Switzerland; 3 Present Address: Department of Quantum Matter Physics, Université de Genève, 1211 Geneva, Switzerland

**Keywords:** Materials for devices, Nanoscale devices

## Abstract

Excellent mechanical properties and the presence of piezoresistivity make single layers of transition metal dichalcogenides (TMDCs) viable candidates for integration in nanoelectromechanical systems (NEMS). We report on the realization of electromechanical resonators based on single-layer MoS_2_ with both piezoresistive and capacitive transduction schemes. Operating in the ultimate limit of membrane thickness, the resonant frequency of MoS_2_ resonators is primarily defined by the built-in mechanical tension and is in the very high frequency range. Using electrostatic interaction with a gate electrode, we tune the resonant frequency, allowing for the extraction of resonator parameters such as mass density and built-in strain. Furthermore, we study the origins of nonlinear dynamic response at high driving force. The results shed light on the potential of TMDC-based NEMS for the investigation of nanoscale mechanical effects at the limits of vertical downscaling and applications such as resonators for RF-communications, force and mass sensors.

## Abstract (web summary)

Suitable materials for nanoelectromechanical systems should possess excellent mechanical properties and allow displacement self-sensing. Here, the authors fabricate electromechanical resonators based on single-layer MoS_2_ with electrical readout, operating in the very high frequency range.

## Introduction

Because of their small mass, nanoscale mechanical resonators have the potential to achieve resonant frequencies and mass sensitivities allowing the detection of single atoms and molecules^1^, beyond those enabled by microelectromechanical systems. However, the small size of nanoelectromechanical systems (NEMS) imposes challenges regarding signal-to-noise ratio and dynamic range^2^, which motivates the investigation of novel materials. Semiconducting two-dimensional transition metal dichalcogenides (TMDCs)^3,4^ with their favorable electrical^5^ and mechanical properties^6,7^, promise new opportunities for the realization of NEMS devices with enhanced output signals.

One of the most often used transduction schemes in NEMS devices, the capacitive transduction scheme, based on the modulation of the capacitive coupling of the resonator to a gate electrode, has been previously applied to multilayer MoS_2_^8–10^. However, since it scales with the area of the resonator, its effectiveness is reduced by the downscaling of resonator dimensions, needed to decrease the resonator mass. An alternative transduction mechanism is readily available for devices based on semiconducting TMDCs. Their band gap is dependent on mechanical strain^11–17^, giving rise to strain-dependent electrical conductivity^18^, which enables the realization of the piezoresistive transduction scheme^19^. In this transduction mechanism, the signal relies on the oscillating strain in the resonator, with smaller lengths resulting in higher strain values for the same amplitude of displacement. Hence, in contrast to capacitive coupling, downscaling is beneficial for piezoresistive transduction. This addresses the low output signal, which is one of the main challenges faced by NEMS resonators. While graphene has been previously shown to perform favorably in the context of NEMS resonators^20^, the absence of a band gap limits the typical piezoresitive gauge factor to ~3 (ref. ^21^), which makes the use of piezoresitive transduction mechanism in this material difficult^22^. In contrast, due to the presence of a band gap, monolayer MoS_2_ has a gauge factor of ~150 (ref. ^18^). Here, we study the dynamic electromechanical response of NEMS resonators based on monolayer MoS_2_ fabricated in the form of resonant channel transistors. Devices show resonance in the range of a few hundred MHz with room-temperature quality factors as high as 300. Analytical and finite element modeling of the resonators’ behavior confirms the dominance of the piezoresistively transduced signal over the capacitively transduced signal. Using the strain-dependent frequency response of the resonators, we extract the resonator mass and built-in strain. The nonlinear dynamic behavior of the resonators is also investigated, demonstrating the influence of nonlinear Duffing force and nonlinear damping. Our findings reveal the potential of atomically thin, monolayer MoS_2_ NEMS resonators for applications such as oscillators for RF communication circuits, mass, and force sensors, as well as the fundamental study of the mechanical degree of freedom and nonlinear dynamics in nanoscale systems.

## Results

### Device characterization

**Fig. 1:**
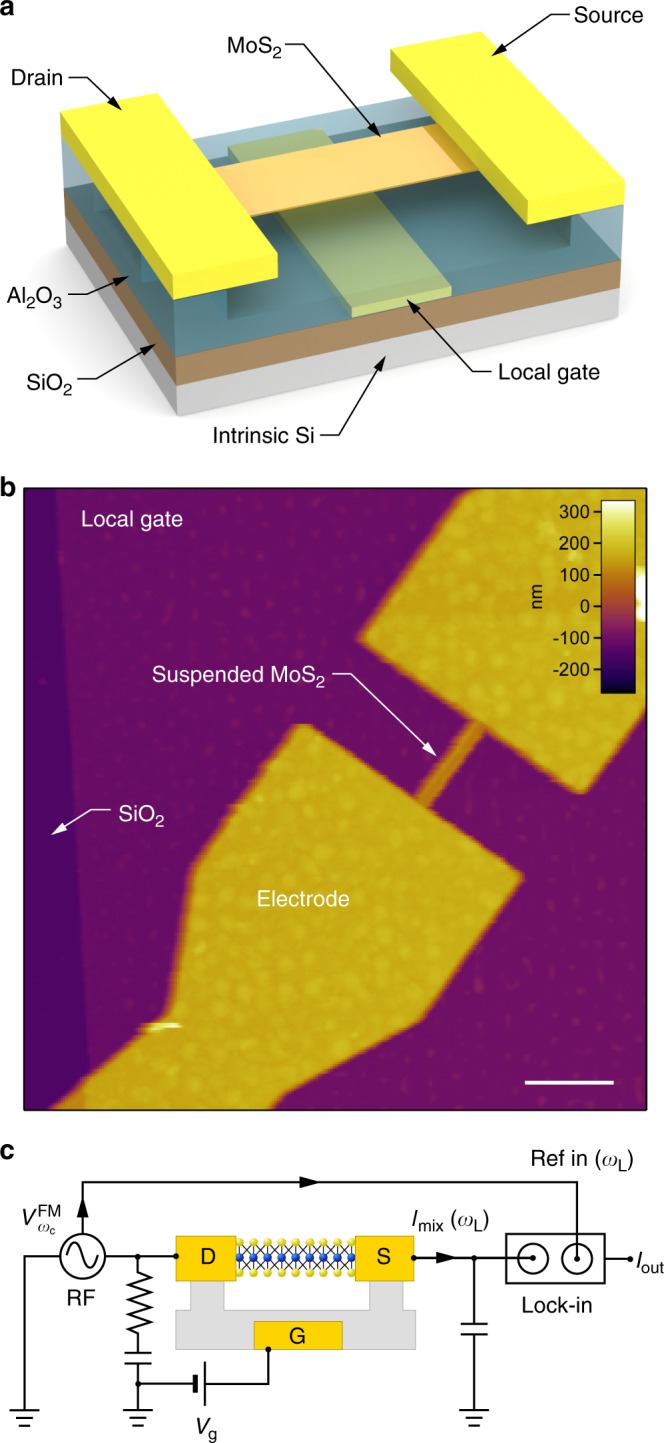
Nanoelectromechanical devices based on monolayer MoS_2_ and the RF electromechanical measurement setup used for characterization of the MoS_2_ NEMS resonators. **a** Schematic of monolayer MoS_2_ resonant channel transistor. The implementation of a local gate helps reducing the parasitic effects for high-frequency measurements. The dielectric underneath the MoS_2_ sheet is etched resulting in suspended contacts in the clamping region. **b** AFM image of a monolayer MoS_2_ ribbon suspended over a local gate electrode and clamped with source/drain electrodes. Scale bar: 1 µm. **c** Schematic illustration of the RF electromechanical measurement setup. A DC voltage is applied to the local gate, and a frequency-modulated voltage $V_{\omega _{\mathrm{c}}}^{{\mathrm{FM}}} = V_{\mathrm{c}}\cos (\omega _{\mathrm{c}}t + \left( {\frac{{\omega _\Delta }}{{\omega _{\mathrm{L}}}}} \right)\sin (\omega _{\mathrm{L}}t))$is applied to the source electrode. The mixing current *I*_mix_ is detected using a lock-in amplifier, locked at the reference frequency *ω*_L_

Monolayer MoS_2_ NEMS were fabricated in the form of suspended devices featuring an embedded local gate, resulting in reduced parasitic capacitance (Fig. 1a). We base our devices on scalable, chemical vapor deposition (CVD)-grown material. The detailed fabrication process is described in Supplementary Note 1. Atomic force microscope (AFM) image of a typical device is shown in Fig. 1b. Figure 1c shows the schematic illustration of the electromechanical characterization setup implementing an all-electrical actuation and detection technique. Basic electrical characterization of the device is presented in Supplementary Fig. 1 and Supplementary Note 2. A direct current (DC) voltage *V*_g_ is applied to the local gate electrode, which induces an electrostatic force between the resonator and the gate, resulting in elastic elongation of the MoS_2_ membrane and static strain in the suspended structure. The resonator is driven by applying a frequency-modulated AC bias voltage$V_{\omega _{\mathrm{c}}}^{{\mathrm{FM}}}$ to the device, giving rise to a time-varying electrostatic interaction between the resonator and the local gate electrode oscillating at the driving frequency *ω*_c_ and resulting in the actuation of the device. The mechanical motion is detected using the mixing technique, which allows the detection of high-frequency motion at lower mixed-down frequencies, thus avoiding issues related to impedance mismatch and parasitic effects, which make measuring the direct response at radio frequencies difficult (Supplementary Note 3). For different values of *V*_g_, the driving frequency is swept over a large spectrum. The resonator acts as a demodulator and yields a mixed-down current *I*_mix_, which is detected using a lock-in amplifier, allowing the detection of nm-scale vibration amplitudes at radio frequencies.

**Fig. 2:**
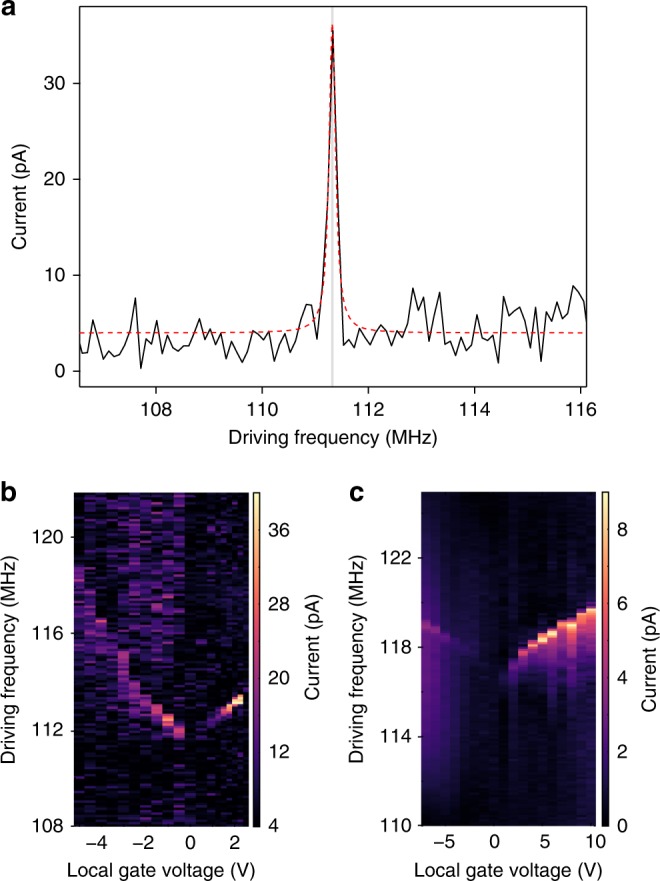
Electromechanical characterization of MoS_2_ NEMS resonators. **a** Frequency response of the mixing current for device R1, showing a characteristic peak around 111 MHz. The extracted quality factor is 225. The measurement is performed at *V*_g_ = 1.5 V and input RF power = −1 dBm. **b**, **c** The frequency response mapped as a function of local gate voltage and driving frequency for resonator R1 in **b** and R2 in **c**. The resonant frequency is highly tunable with gate voltage and shows different curvature, being concave for R1 and convex for R2. The faint background is due to the noise in mixing current. The input RF power is −1 and 8.5 dBm for R1 and R2, respectively

In Fig. 2a we show the room-temperature measurement of *I*_mix_ as a function of driving frequency *ω*_c_ for a resonator with a width of 110 nm and a length of 1.1 µm (Device R1). The mixing current recorded for *V*_g_ = 1.5 V shows a resonance peak near 111 MHz, which corresponds to the mechanical resonance of the MoS_2_ membrane. At low *V*_g_, the driving amplitude is expected to be small and the peak has a characteristic Lorentzian lineshape. We estimate the mechanical quality factor, *Q* = 225, assuming a linear harmonic oscillator^23^. The average value of the quality factor derived from low-drive measurements on seven devices is 190 and the highest quality factor is ~300. These are comparable to quality factors derived from optical readout of monolayer MoS_2_ drumhead resonators^24,25^.

Owing to the presence of a strong piezoresistivity in atomically thin MoS_2_ layers^18^, the mechanical vibrations of the nanoribbon are translated into electrical signals via piezoresistive transduction, in addition to the more traditional capacitive transduction. A detailed comparison of the influence of both transduction mechanisms (Supplementary Note 4) reveals that the contribution from piezoresistive transduction is three times higher than the contribution of the capacitive transduction for our main device. Finite element modeling (Supplementary Note 5) further confirms the dominance of the piezoresistive response.

In Fig. 2b, c, we map *I*_mix_ as a function of the driving frequency and gate voltage for device R1 (described above) and device R2 (width = 215 nm, length = 970 nm). The resonance peak shifts towards higher frequencies with increasing *V*_g_. The modulation of the resonant frequency with the local gate voltage has an electrostatic origin. Monolayer MoS_2_ resonators operate in the membrane limit (Supplementary Note 6), so that in the absence of a local gate voltage, the resonant frequency is defined by the built-in tension. Increasing *V*_g_ results in the appearance of a DC electrostatic force *F*_es_ acting between the suspended device and the local gate electrode. This induces transverse deflection and additional tension on the resonator, increasing the resonant frequency. Therefore, it is possible to tune the resonant frequency using an electrical voltage, similarly to other atomically thin resonators^20,26^.

**Fig. 3:**
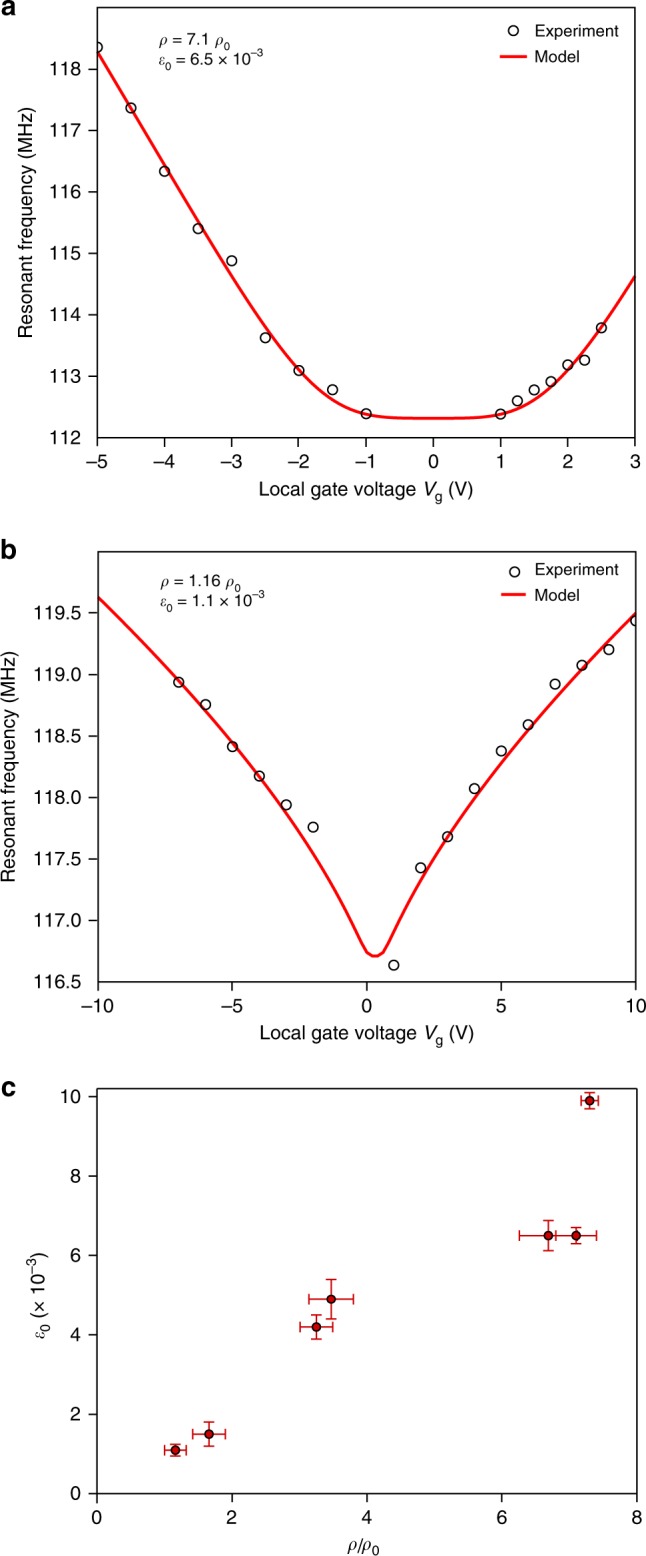
Modulation of the resonant frequency of atomically thin resonators under tensile strain. **a**, **b** Experimentally measured resonant frequency as a function of local gate voltage *V*_g_ (the black markers) for R1 and R2, respectively. The solid red lines are obtained by fitting to the continuum mechanics model for membranes (described in Supplementary Note 6 and ref. ^20^) to determine the values of *ρ*_2*d*_ and *ε*_0_ for each device. As predicted by the model, higher values of built-in strain result in a concave curvature (R1), while the curvature is convex for lower built-in strain (R2). **c** Overview of the extracted mass density and built-in strain for seven MoS_2_ NEMS resonators. The built-in strain is higher for resonators with higher mass density, indicating that the presence of contamination induces strain on the MoS_2_ membrane. Error bars represent SD based on the precision of geometry determination

We use a previously derived model^20^ to describe the gate voltage-dependent resonant frequency (Supplementary Note 6 and ref. ^20^), and fit the model to our experimental results to extract the resonators’ mass density and built-in strain. Figure 3a, b show the resonant frequency as a function of the local gate voltage for device R1 and R2, respectively. We extract the mass density and the built-in strain for each device, resulting in *ρ*_2*d*_ = 7.1*ρ*_0_ and *ε*_0_ = 6.5 × 10^−3^ for R1 and *ρ*_2*d*_ = 1.16*ρ*_0_ and *ε*_0_ = 1.1 × 10^−3^ for R2, with *ρ*_0_ = 3.3 mg m^−2^ representing the mass density of pristine MoS_2_. Mass density values higher than *ρ*_0_ are expected due to the presence of adsorbed residue from the fabrication process, even after thermal annealing in vacuum. Additionally, the resonant frequency shift shows different curvatures for devices R1 and R2 due to different built-in strain, starting with a convex shape for low built-in strain and evolving into a concave curve as the built-in strain increases (Supplementary Figs. 2 and 3).

Figure 3c summarizes the mass density and the built-in strain extracted from measurements on seven resonators. Resonators with higher mass density (more adsorbates) have higher built-in strain, indicating that the presence of contamination is introducing built-in strain on the membrane. In addition to the adsorbates, the presence of built-in tensile strain could be attributed to the top-down fabrication process, resulting in the extension of the suspended membrane with respect to its rest length. Nevertheless, the values of built-in strain are several orders of magnitude lower than the intrinsic strain limit (~11%)^6^.

**Fig. 4:**
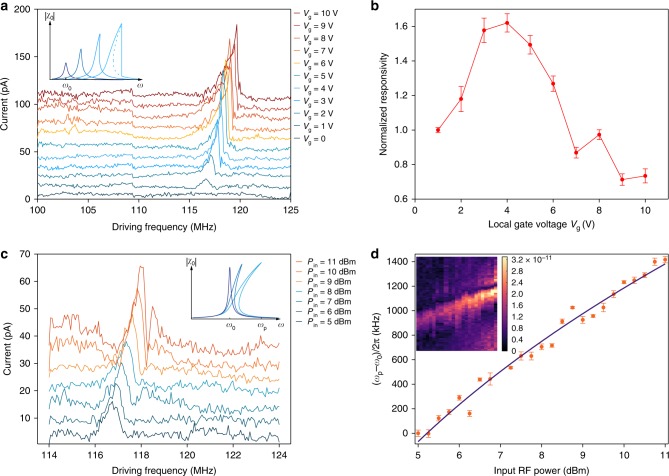
Nonlinear behavior of the MoS_2_ NEMS resonators and the dependence of peak current frequency *ω*_p_ on the input RF power. **a** The frequency response of the mixing current for different values of *V*_g_. Curves are offset for clarity. The onset of nonlinearity occurs at *V*_g_ = 2 V at an amplitude of motion estimated around 1.8 nm (Supplementary Eq. 23). Inset: evolution of the frequency response with increasing *V*_g_. The dashed line shows the solution to the Duffing equation. Due to bistability, the response follows the solid line and drops abruptly. **b** Responsivity of the device defined as the ratio of the peak current to the drive amplitude as a function of local gate voltage for R2. **c** Frequency response of the mixing current for different values of input RF power. Curves are offset for clarity. The inset shows the schematic of the frequency response for increasing *P*_in_. **d** Deviation of the resonant frequency (*ω*_p_ − *ω*_0_)/2*π* as a function of input RF power. The red markers correspond to the experimentally measured values while the black solid line is a fit to the power law $\left( {\omega _{\mathrm{p}} - \omega _0} \right) \propto \left( {P_{{\mathrm{in}}}^{{\mathrm{RF}}}} \right)^{\frac{1}{3}}$. (Supplementary Note 10). The inset shows the measured mixing current mapped as a function the driving frequency (vertical axis) and the input RF power (horizontal axis). The local gate voltage is kept at 1 V throughout the measurements. Error bars represent SD

High-aspect-ratio NEMS resonators are predicted to enter a nonlinear regime at high amplitudes of motion *x* (ref. ^2^). They can, therefore, be more accurately described by the Duffing resonator model^27^, which is effectively a harmonic oscillator model with additional terms (*α/m*) *x*^3^ and $\left( {\eta /m} \right) x^2\dot x$ that correspond to the nonlinear restoring (Duffing) force and nonlinear damping^28^, respectively (Supplementary Note 7). As we increase *V*_g_ in our devices, we observe a change in the resonance peak from a symmetric lineshape to an asymmetric one (Fig. 4a), confirming that MoS_2_ resonators have entered the nonlinear regime. To extract the relevant parameters *α* and *η*, we first consider the frequency response in the absence of nonlinear damping, allowing us to investigate the effects of the nonlinear restoring (Duffing) force (Supplemetary Fig. 4). The resulting frequency response is shown schematically in the inset of Fig. 4a. Increasing the local gate voltage shifts the peak position (via application of electrostatic strain), and also results in the distortion of the peak shape and bistability due to the increasing contribution of the Duffing force as a result of the increased oscillation amplitudes. For device R2, we estimate the Duffing coefficient *α* ~ 1.5 × 10^15^ kg m^−2^ s^−2^ (Supplementary Note 8), which is higher than for carbon nanotube-based devices^26^ and an order of magnitude smaller than for graphene^26^. The lower Duffing coefficient in MoS_2_ as compared to graphene is in line with theoretical calculations^2^ and could be explained by the lower thickness of graphene layers.

The onset of nonlinearity can be seen at *V*_g_ ~2 V. Observation of nonlinearity at such relatively low-drive amplitudes is not surprising since a reduced dynamic range is a well-known drawback of the miniaturization of electromechanical systems. On the other hand, the atomic thickness of MoS_2_ resonators and the fact that they operate in the strain-dominated regime present an opportunity to improve the dynamic range, since the higher built-in strain enhances the dynamic range^2^. We use expressions derived in theoretical studies^2,29–31^ to estimate for device R2 the dynamic range DR ~73.5 dB and the critical amplitude at the onset of nonlinearity *α*_c_ ~1.8 nm, compared with ~60 dB^20^ for graphene-based devices with capacitive readout (Supplementary Note 9).

Next, we consider the effect of nonlinear damping on the frequency response. The effective damping in Duffing resonators is a superposition of linear and nonlinear damping (Supplementary Note 8), with the latter becoming considerable for large amplitudes of vibration. A helpful way to distinguish between the two forms of damping is to look at the responsivity of the resonator defined as the ratio of the peak current to the drive amplitude (Supplementary Note 8, Supplementary Eq. 22). Figure 4b shows the responsivity plotted as a function of gate voltage with values normalized to the responsivity at *V*_g_ = 1V. At low drive, the nonlinear damping is negligible. Therefore, increasing the drive amplitude leads to enhanced responsivity. By further increasing the drive, the motional amplitude increases, resulting in stronger nonlinear damping and, therefore, stronger effective damping of the resonator. As a result of the increased effective damping, the responsivity as a function of applied gate voltage becomes flat at *V*_g_ ~4 V and eventually decreases as the drive amplitude becomes larger.

Resonance peaks also broaden in the nonlinear regime (Supplementary Fig. S4c). This is consistent with reports on resonators based on graphene and carbon nanotubes^26^, confirming that nonlinear damping is a robust phenomenon in resonators with atomic-scale transverse dimensions. Various sources of nonlinear damping have been suggested, including nonlinear dissipation mechanisms (e.g., due to friction with the clamps^32^), as well as the effect of geometrical nonlinearities on dissipation channels, which could be internal to the resonator or external (contamination or clamping losses)^26,33,34^.

Another approach to increasing the driving force and amplitude of motion is to increase the input RF power *P*_in_. Figure 4c shows the resonant response for device R2 for different *P*_in_, with higher input power resulting in a stronger influence of nonlinear effects on the peak shape. Consequently, the frequency corresponding to the maximum of mixing current (*ω*_p_) deviates from the frequency of the linear harmonic oscillator (*ω*_0_) (Supplementary Note 10). The frequency shift Δ*ω*/2*π* = (*ω*_p_ – *ω*_0_)/2*π* as a function of input RF power is shown in Fig. 4d, with the fit to the experimental data confirming that Δ*ω* is proportional to (*P*_in_)^1/3^ (Supplementary Note 10). The frequency shift Δ*ω* with increasing *P*_in_ originates from effects different from the shift in the resonant frequency induced by the static strain, previously shown on Fig. 3b. Here, increasing *P*_in_ does not contribute to the static strain due to the fact that the average electrostatic force from the RF voltage is zero. Instead, the peak of the frequency response is pulled towards higher frequencies due to the presence of Duffing nonlinearity and the time-varying tension induced by a sufficiently large vibration amplitude as a result of increasing *P*_in_. These two origins are illustrated in the insets of Fig. 4a, c.

## Discussion

We report on the response of single-layer MoS_2_ electromechanical devices to dynamic mechanical stimulation. We show that the presence of piezoresistivity in MoS_2_^18^ provides an alternative transduction mechanism that enhances the output electrical signal of MoS_2_ NEMS resonators. Our atomically thin resonators operate in the very-high-frequency range with resonant frequencies that are predominantly defined by the built-in strain and are tunable using a voltage applied between the gate electrode and the membrane. Additionally, our results show that nonlinear effects play an important role in the dynamic behavior of MoS_2_ NEMS resonators and must be taken into account when designing resonators for a target dynamic range. This study demonstrates the promise of MoS_2_ resonators for integration in high-sensitivity mass and force sensors, low-footprint oscillators, as well as their potential for the fundamental study of the mechanics at the interface between the quantum and classical regime.

## Methods

### MoS_2_ growth

Monolayer MoS_2_ is grown by CVD on *c*-plane sapphire using a gas-phase reaction between MoO_3_ (≥99.999% Alfa Aesar) and high-purity sulfur evaporated from the solid phase (≥99.99% purity, Sigma-Aldrich)^35^. We use Raman spectroscopy to confirm the monolayer thickness of the grown MoS_2_ film (Supplementary Fig. 6).

### Transferring MoS_2_ from the growth substrate

CVD-grown single domains of MoS_2_ were transferred from sapphire onto embedded local gates covered with 250 nm of Al_2_O_3_, using the KOH transfer method. Samples were first spin coated with poly(methyl methacrylate) (PMMA) 950 MW 2% in anisole at 1500 r.p.m. to obtain a ∼100-nm-thick polymer film. Next, the coated substrates are left in vacuum for 12 h to remove the solvent. We avoid baking the resist to prevent the potential build-up of mechanical stress. The sapphire substrate was etched in KOH (30%) at moderate temperatures (60–70 °C), resulting in detached CVD material on top of the PMMA support film. Films are washed several times in deionized water, and transferred on top of the embedded local gates and dried at 40 °C for 1 h. The polymer is dissolved in acetone, and the flakes are cleaned from residues by annealing at 250 °C in argon/hydrogen atmosphere for 8 h.

### Fabrication of resonant channel transistors

After transferring CVD-grown MoS_2_ on top of local gates, the single crystalline MoS_2_ domains are etched into nanoribbons with a width varying between 80 and 220 nm, followed by deposition of metal electrodes on the top of the nanoribbons. In the last step, the mechanical degree of freedom is attained by releasing the MoS_2_ channel in a wet etching process followed by critical point drying. The effect of the suspended contacts can be easily identified in the mechanical response due to their lower quality factor and lack of sensitivity to the gate voltage (Supplementary Fig. 7 and Supplementary Note 11).

## Supplementary information

Supplementary Information

## Data Availability

The data that support the findings of this study are available from the corresponding author on reasonable request.
