# Supplementary material for: Self-sensing, tunable monolayer MoS2 nanoelectromechanical resonators
Source: Nat Commun. 2019 Oct 23;10:4831. doi: 10.1038/s41467-019-12795-1 (PMC6811529; doi:10.1038/s41467-019-12795-1)
Supplement: Supplementary file 1 — Supplementary Information [file 41467_2019_12795_MOESM1_ESM.pdf]

Supplementary Information

for

Self-sensing, tunable monolayer MoS_2 nanoelectromechanical resonators

Sajedeh Manzeli et al.

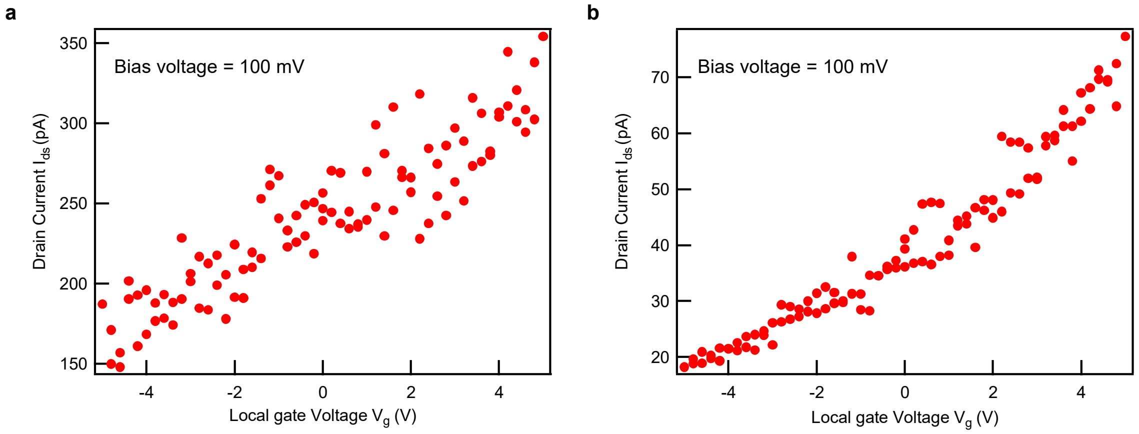

Supplementary Figure 1. DC transfer characteristics of the suspended-channel monolayer MoS_2 transistors with local gate electrodes. (a) Device R1 and (b) Device R2. Within the range of applied gate voltage, the device remains in the subthreshold regime.

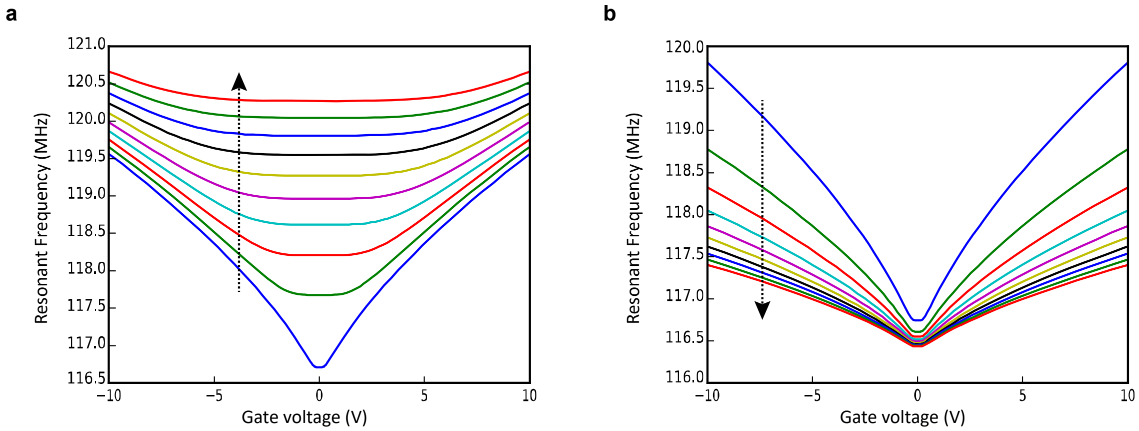

Supplementary Figure 2. Analytically derived gate voltage induced resonant frequency tuning based on the continuum mechanical model. (a) Resonant frequency plotted as a function of gate voltage for various amounts of built-in strain. The arrow shows the direction of increasing built-in strain. We note that increasing the built-in strain results in higher $f_0(\varepsilon_0, \rho_{2d})$ while the sensitivity to the applied gate voltage decreases, which is expected as the electrostatically induced strain becomes a smaller contribution to the total strain in comparison to the built-in strain. Additionally, the curvature of the tuning curve changes from convex shape for small built-in strain to a concave shape as the built-in strain increases. (b) Resonant frequency plotted as a function of gate voltage for increasing mass density (the direction of the arrow). The higher the mass density, the lower $f_0(\varepsilon_0, \rho_{2d})$ would be and the tunability decreases. In contrast to the effect of built-in strain, the curvature of the tuning curve does not change for higher mass density.

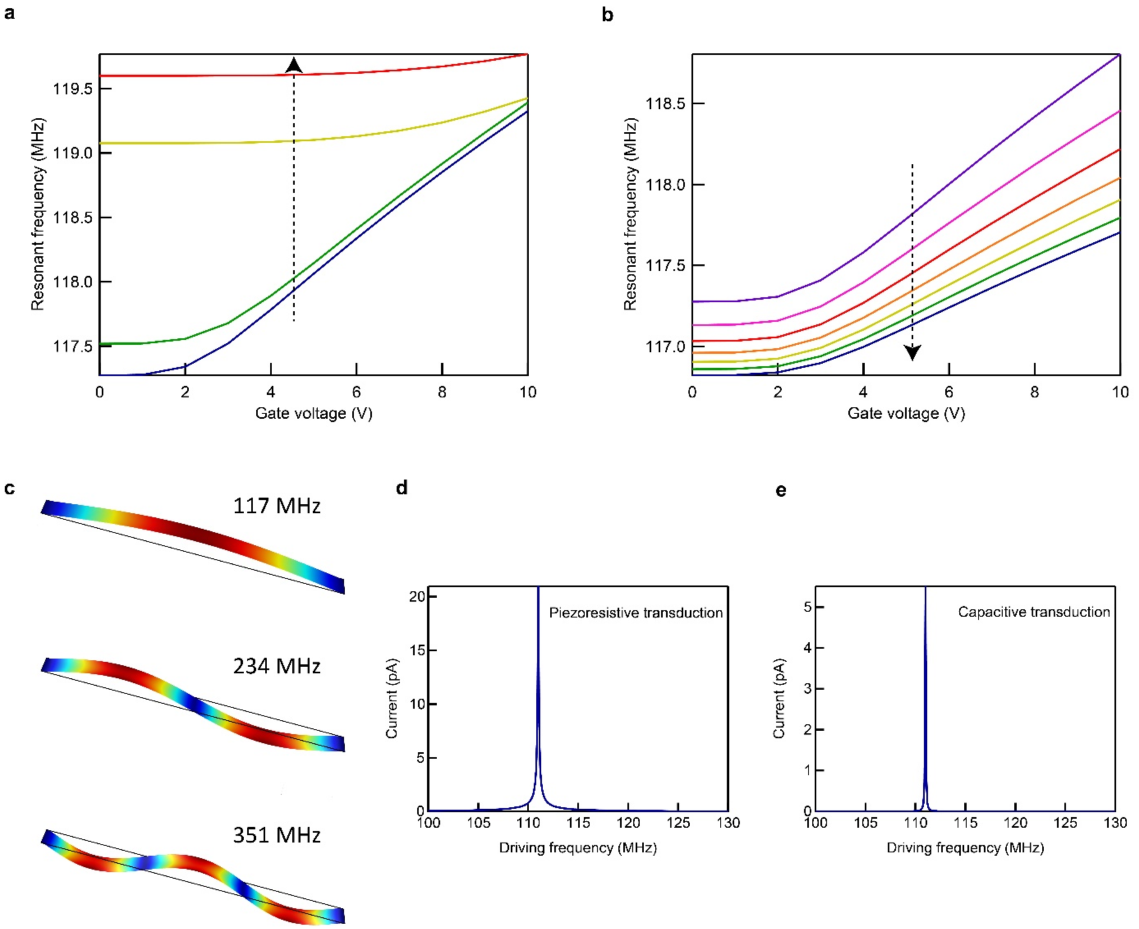

Supplementary Figure 3. Finite element modeling of the dynamic behavior of the MoS_2 NEMS resonators. (a) and (b) Gate voltage induced tuning of the resonant frequency. In order to compare the results with Supplementary Figure 2 the model is built for a resonator with the same dimensions as R2. The effect of increasing built-in strain (a) and mass density (b) on the tuning curve is investigated. The direction of the arrows show the increase in the built-in strain and mass density. (a) For higher built-in strain the resonant frequency at zero gate voltage increases. The higher the built-in strain, the lower the sensitivity to the applied gate voltage. Also, the curvature of the tuning curve changes with increasing the built-in strain. (b) Resonator with a higher mass density have a lower resonant frequency, and, the heavier the resonator gets, the less sensitive it becomes to the applied gate voltage. The curvature of the tuning curve is independent of the mass density. (c) The mode shapes and resonant frequencies of the first three flexural modes for the same geometry as R2. (d) and (e) show the FEM simulation of the piezoresistive and capacitive transduction of mechanical vibrations. In order to compare with the calculation in Supplementary Note 4, we modeled resonators with the geometry, built-in strain and mass density as extracted for device R1. The results show that the piezoresistive transduction mechanism is expected to be a factor of 4 stronger than the capacitive transduction mechanism.

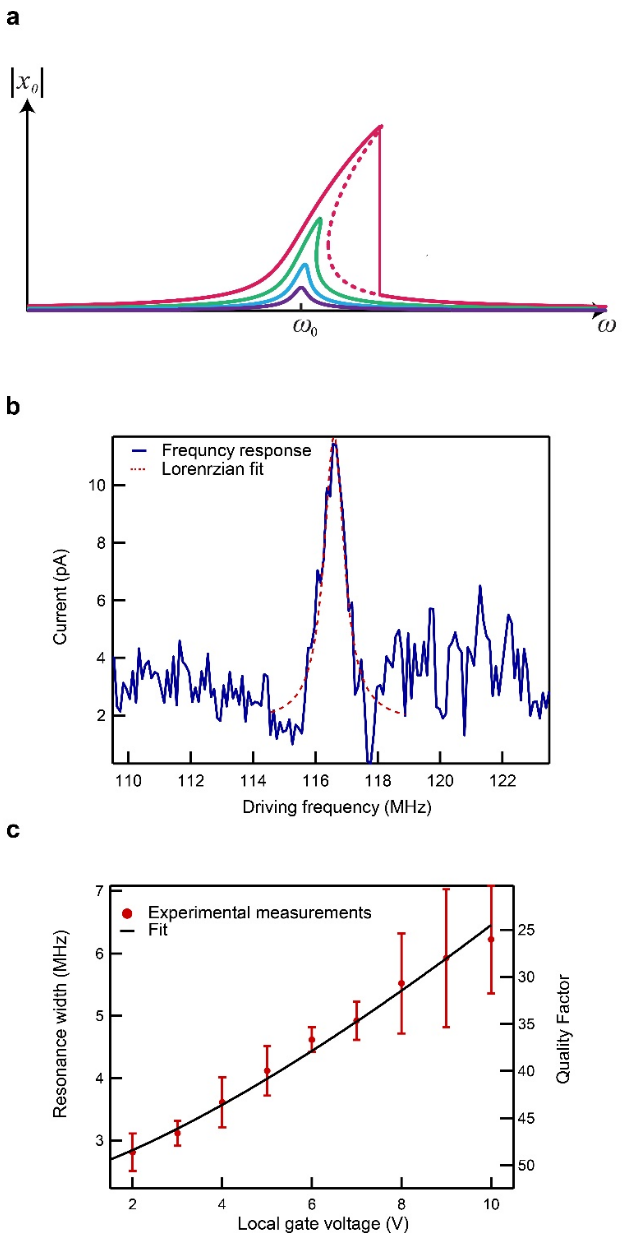

Supplementary Figure 4. The effect of nonlinear restoring force on the frequency response of doubly clamped MoS_2 resonators, and the dependence of resonant width on the nonlinear damping mechanism (a) Schematic illustration of the frequency response of a Duffing resonator for $\eta = 0$. For the small drive the peak has a symmetric shape with a maximum at ω_0 (frequency of linear harmonic oscillator). As the drive increases, the peak shape becomes asymmetric. The schematic illustration of the response corresponding to the highest drive (red), illustrates the appearance of bistability in the frequency response. (b) The frequency response of the mixing current for device R2 from the main text. The local gate voltage $V_g = 1$ V. The dashed red line shows the Lorentzian fit to the resonant peak. The extracted values of resonant width and quality factor are 1.6 MHz and 75, respectively. (c) The resonant width and the quality factor as a function of local gate voltage, measured for asymmetric peaks of device R2 (Figure 4a from the main text). The red markers are the experimental results and the black line is a fit to the model described by Eichler et al.[12]

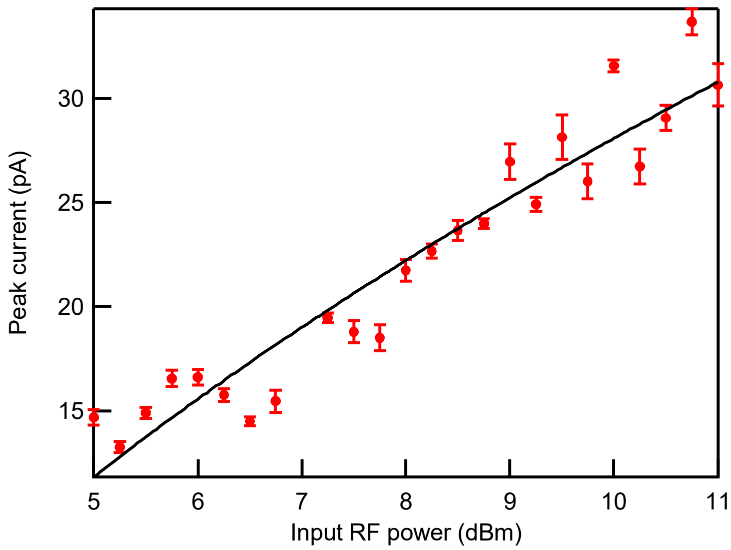

Supplementary Figure 5. Peak current as a function of input RF power. The experimentally measured (red markers) peak current increases with the input RF power, and is proportional to the square root of the input RF power (black line).

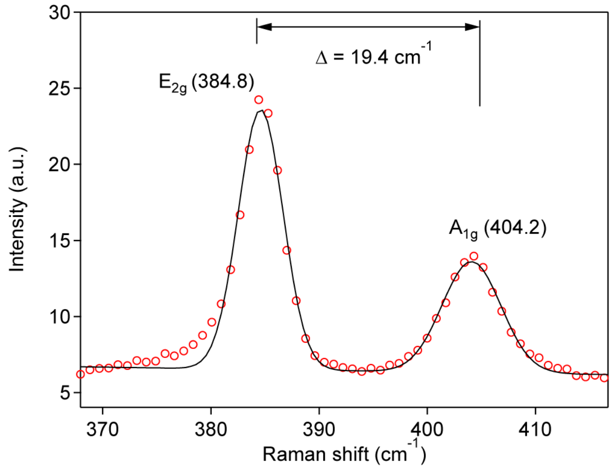

Supplementary Figure 6. Raman characterization of monolayer MoS_2. Raman spectra of transferred MoS_2 from a single crystal in the vicinity of the device presented in the main manuscript, using a 532 nm laser excitation and a 3000 line mm^{-1} grating. The observed difference between E_{2g} and A_{1g} Raman modes of MoS_2 is consistent with a monolayer.

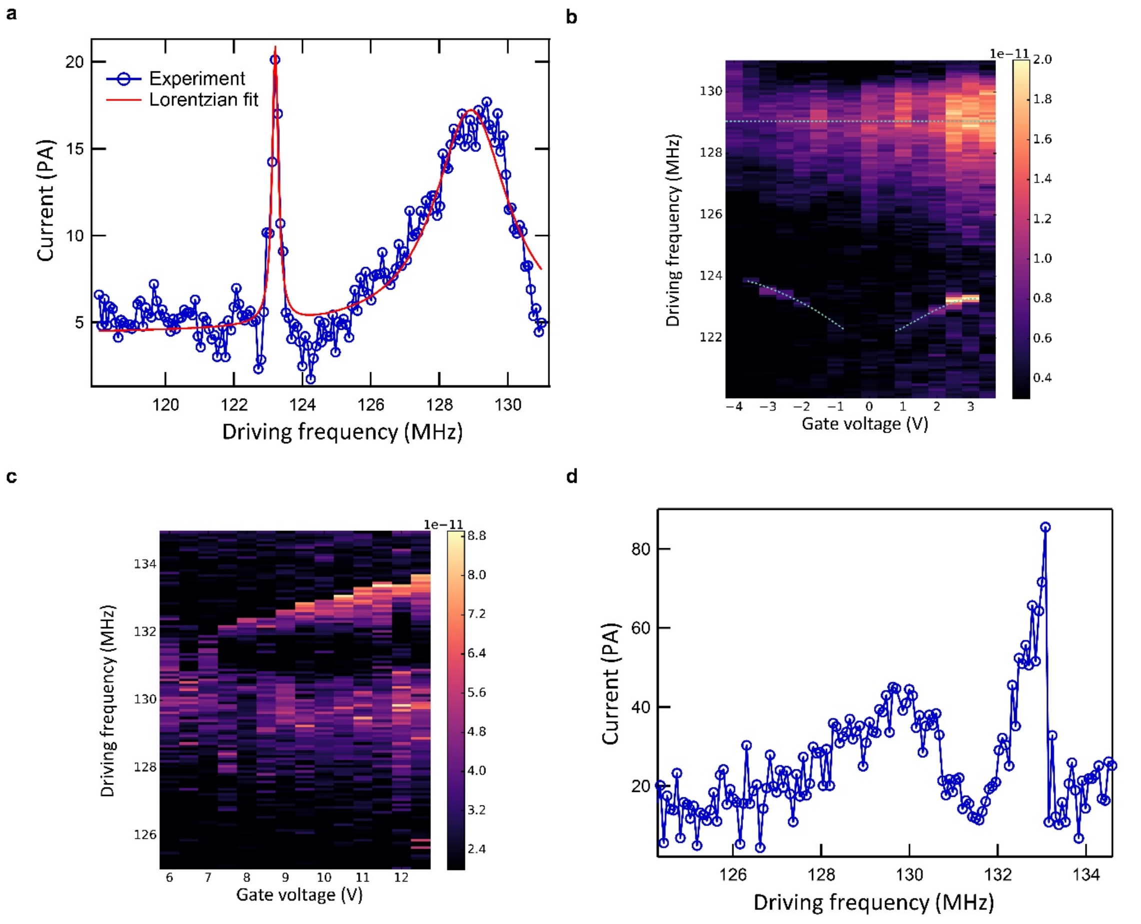

Supplementary Figure 7. Vibrations of the suspended metal contacts. (a) Resonant peaks from the vibrations of the MoS_2 membrane (~ 123 MHz) and the vibrations of the suspended part of the metal contacts (~ 129 MHz), measured at $V_g = 2.5$ V. Mechanical resonance of the MoS_2 membrane results in a much narrower peak ($\Delta\omega \sim 0.6$ MHz) in comparison with the width of the peak originating from the suspended metal contacts ($\Delta\omega \sim 5$ MHz). (b) The mixing current mapped as a function of the driving frequency and the local gate voltage. The dashed lines are guides to the eye. The resonant peak from the monolayer MoS_2 membrane is defined by the applied tension, and thus, is tunable by changing the applied electrostatic strain. In contrast, the resonant behavior of the thick metal contacts is in the plate limit, and therefore, the resonant frequency is independent of the applied tension (local gate voltage). (c) Further increasing the local gate voltage increases the resonant frequency of the MoS_2 vibrations to surpass the resonant frequency of the suspended contacts. For gate voltages up to 12.5 V the resonant frequency of MoS_2 membrane remains tunable and the resonant frequency of the metal contacts remains independent of the applied electrostatic strain. (d) The frequency response of the mixing current for $V_g = 10.5$ V. The resonant peak from the MoS_2 membrane is now at a higher frequency compared to the resonant frequency of the suspended contacts. Additionally, as a result of high drive amplitude due to the high V_g, the peak becomes asymmetric which confirms that the MoS_2 membrane has entered the nonlinear regime.

Supplementary Note 1: Device fabrication

We have fabricated suspended channel MoS_2 devices featuring a local bottom gate. The presence of local gate minimizes the overlap area between the gate and the source/drain electrodes, and reduces the source-gate and the drain-gate capacitances. This is important since at higher frequencies the impedance of the parasitic capacitances between the gate and the source/drain electrodes are no longer negligible and could introduce filtering effects on the output electrical signal.

The fabrication starts by depositing local gate electrodes on an intrinsic Si substrate covered by 270 nm of oxide. Subsequently, 250 nm of Al_2O_3 is deposited on top of the gate electrodes, using atomic layer deposition (ALD), which acts as the gate dielectric. In the next step, single crystalline domains of CVD grown monolayer MoS_2 are transferred on top of the local gates. The material synthesis is described elsewhere.[1] Using CVD materials allows controlling the reproducibility of device properties and increases the throughput of the fabrication process.

The MoS_2 flakes were then shaped into nanoribbons using a polymethyl methacrylate (PMMA) mask patterned in an electron-beam lithography (EBL) step followed by a reactive ion etching process in oxygen plasma and mask removal in acetone. Metal electrodes (Cr/Au) were deposited by standard bilayer technique (methyl methacrylate (MMA)/PMMA) in electron-beam lithography (EBL), followed by thermal evaporation and lift-off in acetone. The metal contacts where then thermally annealed in Ar at 200 ºC for 2 hours.

Wet etching using orthophosphoric acid undercuts the Al_2O_3 to yield suspended MoS_2 channel. The suspended devices were successively transferred to water and ultrapure ethanol, followed by critical point drying (CPD) to prevent the suspended structure from collapsing due to capillary forces and surface tension. Prior to electromechanical measurements, samples are imaged using atomic force microscope (AFM) in tapping mode to verify their suspension. (Figure 1b from the main text).

Supplementary Note 2: DC transport characterization of the devices

Following the realization of suspended structure, the resonators are bonded on a chip carrier and loaded in high vacuum where all the electrical/electromechanical characterization is carried out. The devices are then annealed at 170°C for more than 24 hours to remove water and other adsorbates from both surfaces of the suspended 2D channel.

After annealing and prior to RF electromechanical measurements, we characterize the electrical transport behavior of resonant channel transistors in the DC regime using Agilent E5270B parameter analyzer. Supplementary Figure 1 shows the DC transfer characteristics for devices R1and R2 from the main text.

The transfer characteristics show a relatively low conductivity which is expected since the devices are operating in the subthreshold regime. We avoid higher values of gate voltage to prevent the collapse of suspended structure before electromechanical characterization.

Supplementary Note 3: The RF electromechanical characterization setup

During the DC characterization of the suspended channel transistors, the chip carriers are already mounted on a holder which is connected to SMA connectors via a printed circuit board. The RF signals are transmitted using high frequency coaxial cables and all the connectors on the cables and vacuum feedthrough are of the SMA type with a frequency range up to 3 GHz and a voltage standing wave ratio (VSWR) of less than 1.3. We used as a microwave signal

generator Keysight N5183B and the mixed-down current was detected using the current input of a Stanford Research Systems SR830 DSP lock-in amplifier. The DC gate voltage was applied using a Keithley 2450 source measure unit. The matching load on the signal input consists of a 50 Ω resistor and 100 pF capacitor connected in series. The 100 nF capacitor connected to the signal output provides an RF ground and eliminates fluctuations in the frequency response of the mixing current due to the floating output. Figure 1c in the main text shows the circuit diagram for the RF measurement setup.

Detection technique

The frequency response is detected indirectly, using a mixing technique which facilitates the detection by transferring the output signal to lower frequencies without the loss of the information stored in its amplitude. To implement the signal mixing we choose frequency modulation (FM) over amplitude modulation (AM) or 2-source techniques, because FM only detects the terms with electromechanical origin and suppresses the purely electrical terms.[2] Another advantage of FM or AM or 2-source technique is that the absence of background signal results in enhanced signal-to-background ratio and signal-to-noise ratio.[2] We apply to the source electrode, an FM modulated signal in the form of $V_{\omega_c}^{FM}(t) = V_c\, cos(\omega_c t + \left(\frac{\omega_\Delta}{\omega_L}\right) sin(\omega_L t))$ where V_c is the amplitude of the applied voltage, ω_c is the carrier frequency in the RF range, t is time, $\omega_\Delta\, /2\pi$ is the frequency deviation (typically 50 kHz to 100 kHz), and $\omega_L/2\pi$ (typically 1 kHz) is the reference frequency at which the demodulated signal is detected using the lock-in technique.

Frequency deviation ω_Δ is proportional to the amplitude of the detected signal and could be adjusted to get a reasonable signal to noise ratio. On the other hand, high values of ω_Δ result in the contributions from higher-order terms to be detected in the measured signal[2] which leads to nonlinearity and broadening of the peak shape that does not originate from the physics governing the mechanical behavior of the resonator, but is due to the nonlinearities in the actuation and detection mechanisms. Additionally, it is crucial to choose values of ω_Δ and ω_L that result in a transient regime that disappears before being detected. This allows detection of the steady state response which is the regime of interest in the behaviour of NEMS resonators. Both concerns could be addressed by choosing ω_L and ω_Δ that are much smaller than the resonant width $\Delta\omega$.[2] This is especially important for studying the nonlinear behavior of our resonators in the absence of contributions from additional sources of nonlinearity that come from the experimental detection technique. In practice, it is possible to find the appropriate range for ω_Δ by monitoring the evolution of the peak shape with increasing ω_Δ.[2]

Supplementary Note 4: Comparison between piezoresistive transduction and electrostatic transduction

When using FM detection, the source-drain current I_{ds} is composed of several high-frequency components ($\omega_c, 2\omega_c, \ldots$), a DC component and a mixing component at ω_L, with the latter being the only electromechanical term in I_{ds} and the only term that will be detected by the lock-in amplifier.

The mixed-down current at frequency ω_L is proportional to $|\partial x/\partial\omega|$, with x the amplitude of the mechanical motion of the resonator. Gouttenoire et. al. have developed the mathematical basis for determining the electromechanical current detected by the FM technique (I_{mix}^{FM}), showing that[2]:

$$I_{mix}^{FM} = \frac{1}{2}\frac{\partial G}{\partial x} V_c \frac{\partial x}{\partial \omega}\omega_\Delta \cos{(\omega_L t)} \quad (1)$$

where $G = \frac{\partial I_{\mathrm{ds}}}{\partial V_{\mathrm{ds}}}$ is the conductance of the device and $\frac{\partial G}{\partial x}$ translates the mechanical vibrations into an electrical signal and, therefore, it is defined by the transduction mechanism.

The traditional capacitive transduction relies on the capacitive coupling between the suspended membrane and the local gate electrode. The vibrations modulate the gate capacitance (C_{g}), which in turn modulates the charge carrier density in the semiconducting MoS_2 channel and results in the modulation of the conductance. Because of the oscillating conductance, the mechanical vibrations of the resonator is transduced into electrical signals. Therefore, the capacitive transduction mechanism $\left.\frac{\partial G}{\partial x}\right|_{Cap}$ can be formulated as:

$$\left.\frac{\partial G}{\partial x}\right|_{Cap} = \frac{\partial G}{\partial C_{\mathrm{g}}} \frac{\partial C_{\mathrm{g}}}{\partial x} \tag{2}$$

Considering that:

$$G = V_{\mathrm{g}} \frac{C_{\mathrm{g}}}{A} \mu \frac{W}{L} \tag{3}$$

where $A = WL$ is the membrane area and μ is the charge carrier mobility, and since V_{g} is constant for the vibrating membrane:

$$\frac{\partial G}{\partial C_{\mathrm{g}}} = \frac{V_{\mathrm{g}} \mu}{L^2} \tag{4}$$

We estimated the field-effect mobility from the DC transfer characteristics of the device R1 (Supplementary Figure 1a) to be ~ 0.42 cm^2 V^{-1}s^{-1}. This relatively low value of mobility is expected since the device is operating in the sub-threshold regime for the range of V_{g} used to measure the transfer characteristics. We avoided higher values of V_{g} for DC characterization in order to minimize the risk of membrane collapsing due to the electrostatic force from the gate. Using the extracted μ and $\frac{\partial C_{\mathrm{g}}}{\partial x} \sim 17$ pF m^{-2} (derived assuming a parallel plate capacitance), we can estimate for the measured peak presented in Figure 2a in the main text, $\left.\frac{\partial G}{\partial x}\right|_{Cap} \sim 8.8 \times 10^{-4}$ S m^{-1}.

Alternatively, thanks to the presence of strong piezoresistivity in monolayer MoS_2[3], an additional transduction mechanism is available in our resonators. Because of the geometry of a doubly clamped thin membrane, the mechanical vibrations induce oscillating strain due to the periodic elongation of the membrane. The oscillating strain in turn modulates conductivity of the MoS_2 membrane, meaning that, the piezoresistive transduction mechanism $\left.\frac{\partial G}{\partial x}\right|_{\mathrm{PR}}$ can be extracted from:

$$\left.\frac{\partial G}{\partial x}\right|_{\mathrm{PR}} = \frac{\partial G}{\partial \varepsilon} \frac{\partial \varepsilon}{\partial x} \tag{5}$$

with ε being the strain induced by the mechanical motion x of the resonator. From the definition of the piezoresistive gauge factor γ:

$$\frac{\partial G}{\partial \varepsilon} = G_{\mathrm{i}} \times \gamma \tag{6}$$

where $G_{\mathrm{i}} \sim 3$ nS is the initial conductance of the MoS_2 channel when it is not vibrating and is estimated from the DC transport characterization. We approximate the shape of the first mode with two straight lines resulting in $\frac{\partial \varepsilon}{\partial x} \sim \frac{4x}{L^2}$. Using the average value of γ for monolayer MoS_2[3]

and x equal to the vibration amplitude at the onset of nonlinearity (Supplementary Note 10), we derive $\left.\frac{\partial G}{\partial x}\right|_{\mathrm{PR}} \sim 3 \times 10^{-3}$ S m^{-1}.

We find that for R1, the piezoresistive transduction is more than 3 times stronger than the capacitive transduction:

$$\frac{\left.\frac{\partial G}{\partial x}\right|_{\mathrm{PR}}}{\left.\frac{\partial G}{\partial x}\right|_{\mathrm{Cap}}} \sim \frac{G_{\mathrm{i}}\gamma \times 4x}{V_{\mathrm{g}}\,\mu \times \partial C_{\mathrm{g}}/\partial x} \sim 3.4 \tag{7}$$

The piezoresistive transduction mechanism is not available in semimetallic graphene where the piezoresistive gauge factor is two orders of magnitude smaller compared to MoS_2 due to the absence of a bandgap.[4]

Supplementary Note 5: Finite element modeling of atomically thin MoS_2 resonators

Finite element models are built using COMSOL 5.3 to investigate the dynamic mechanical behavior of MoS_2 membranes and the effects of built-in strain, mass density and electrostatically induced strain (via an external gate voltage). Additionally, we simulated the electrical response of the MoS_2 NEMS resonators to compare the piezoresistively transduced response with the capacitively transduced one.

A multiphysics simulation is implemented that would allow the study of the interplay between electrostatic actuation, static and dynamic mechanical response, as well as, the electrical currents resulting from the transduction of dynamic mechanical response.

Devices R1 and R2 are modeled as doubly clamped membranes suspended over an airgap. The membrane geometry is extracted from the AFM topography image in non-contact mode. The AFM images also give the depth of the airgap making it possible to implement the equivalent gate capacitance corresponding to the thicknesses of air dielectric and the remaining Al_2O_3 dielectric.

The material parameters used for MoS_2 are Poisson's ratio ν = 0.27 and Young's modulus E_{Y} = 270 GPa.[5] It is important to notice that even though there is resist residue on top of the MoS_2 membrane, the Young's modulus of MoS_2 is at least two orders of magnitude larger than that of polymer resists and, therefore, it dominantly defines the equivalent stiffness for the combination of MoS_2 and resist adsorbates. The relative dielectric constants of 1 and 9 are used for air and for Al_2O_3, respectively.[6]

MoS_2 is modeled as an isotropic linear elastic material since the applied strain levels are below the range where the nonlinear elastic behavior becomes considerable.[5] Dielectrics are modeled as linear elastic dielectrics with the airgap defined as a nonsolid material that can deform in the direction normal to the membrane. The membrane is meshed using maximum element size of 20 nm in the in-plane direction and 0.5 nm in the vertical direction. The dielectric material is meshed using maximum element size of 20 nm in the in-plane direction and 50 nm in the vertical direction.

The mechanical boundary conditions are set in a way that the membrane is clamped at the two edges. The lower boundary of the dielectric material is connected to a voltage terminal which applies a DC gate voltage. A small-signal perturbative voltage is applied between the clamped edges of the membrane to simulate the AC source-drain voltage.

In order to study the effect of built-in strain, mass density and electrostatically induced strain, we use an electromechanics multiphysics (solid mechanics and electrostatics) package and solve the model with a stationary analysis followed by an eigenfrequency analysis.

From the stationary study, we determine the static vertical deflection and the static strain distribution induced by the electrostatic DC force. The static strain obtained from FEM simulations is in agreement with the analytically calculated strain when approximating the shape of the first mode with two straight lines:

$$\varepsilon_{\mathrm{static}} = \frac{\Delta L}{L} = \sqrt{1 + \left(\frac{2d_{\mathrm{static}}}{L}\right)^2} - 1 \tag{8}$$

here $\varepsilon_{\mathrm{static}}$ is the static strain and d_{static} is the static deflection due to electrostatic force from the DC gate voltage. The eigenfrequency analysis then determines the frequency of each mode as a function of the applied gate voltage, the built-in strain and the mass density.

Investigating the effect of applied gate voltage on tuning the resonant frequency is achieved by solving the model while running sweeps over a given range of gate voltages. In order to compare with the tuning curves obtained from the analytical continuum mechanics model (Supplementary Figure 2), we simulate the frequency tuning for a device with similar dimensions as R2 and record the gate-dependent resonant frequency for different built-in strain values and mass densities. The results are presented in Supplementary Figure 3a and Supplementary Figure 3b and their agreement with the results from the analytical model (Supplementary Figure 2), further confirms the validity of the model and our analysis. Supplementary Figure 3c shows the frequency and the mode shape for the first 3 flexural modes for the model corresponding to device R2 with built-in strain $1.1 \cdot 10^{-3}$ and density of 1.16 ρ_0.

Next, we simulate the piezoresistive and capacitive transduction mechanisms by adding electric currents to the above mentioned multiphysics. The model is, first, solved using a prestressed analysis to obtain the stationary response to the DC gate force. Once the stationary response is obtained a frequency domain analysis solves the dynamic behaviour for a defined frequency range.

To simulate the piezoresistive transduction, the conductivity of the membrane at each point is defined by the strain distribution according to:

$$\sigma = \frac{\sigma_0}{1 + \gamma\, \varepsilon} \tag{9}$$

here σ_0 is the conductivity of the membrane when it is not vibrating, γ is the piezoresistive gauge factor for monolayer MoS_2,[3] ε is the mechanical strain induced by the vibrations of the membrane and is the parameter that couples the mechanical and electrical parts of the multiphysics package.

On the other hand, the capacitive transduction mechanism is modelled by defining the conductivity of the membrane as:

$$\sigma = V_{\mathrm{g}} \mu \frac{C_{\mathrm{g}}}{LWt} \tag{10}$$

here C_{g} represents the capacitive coupling to the gate terminal which is modulated with the mechanical vibrations of the membrane and is the parameter that couples the mechanical and electrical parts of the multiphysics package.

By implementing the models corresponding to each transduction mechanism, we simulate the vibration induced modulation of conductivity. In the case of piezoresistive transduction, the conductivity changes periodically as a result of vibration-induced strain, while in capacitive

transduction the vibrations change the gate capacitance which in turn changes the charge carrier density in the channel.

The solution to the frequency domain analysis determines the current density. The total current is derived by integrating the current density over the cross section of the device. In order to compare the results with the calculations in Supplementary Note 4, the simulated frequency response of device R1 is shown in Supplementary Figure 3d and Supplementary Figure 3e for the piezoresistive transduction and capacitive transduction, respectively. The simulations predict that the piezoresistive transduction is ~4 times stronger than the capacitive transduction, which is in line with the results obtained by analytical estimations in Supplementary Note 4.

Supplementary Note 6: Strain-modulated electromechanical response of the atomically thin MoS_2 resonators

The voltage applied across the gate capacitance results in the application of a force on the suspended resonator which is written as:

$$F = -\frac{1}{2}\frac{\partial C_g}{\partial x}\left(V_g - V_{sd}\right)^2 \quad (11)$$

where $\frac{\partial C_g}{\partial x}$ is the spatial derivative of the gate capacitance, V_g is the static voltage applied to the local gate electrode and V_{sd} is the AC voltage applied between the source and drain electrodes of the device. Linearization of the force for small V_{sd} results in $F = F_{es} + F_{drive}$ where F_{es} is the electrostatic DC force applied on the membrane and is described as:

$$|F_{es}| = \frac{1}{2}\frac{\partial C_g}{\partial x}V_g^2 \quad (12)$$

and F_{drive} is the driving force that actuates the mechanical vibrations and is described as:

$$|F_{drive}| \sim \frac{\partial C_g}{\partial x}V_g V_{sd} \quad (13)$$

While the electrostatic force results in a static deflection of the membrane from its rest position, the driving force leads to a time-varying bending profile.

The dynamic behavior of resonators can be modeled using a common approximation to the equation of motion for a doubly clamped resonator, given by the Euler-Bernoulli Equation[7]. A perturbative treatment of the Euler-Bernoulli equation results in a continuum mechanics model for a doubly clamped resonator with the resonant frequency can be described as[7,8]:

$$f_0 = \frac{\pi}{2L^2}\sqrt{\frac{E_Y t^2}{12\rho} + \frac{T_0 L^2}{\pi^2 \rho W t}} \quad (14)$$

Where f_0 is the resonant frequency of the fundamental mode, L, W and t are the length, width and thickness of the resonator, E_Y is the Young's modulus, T_0 is the built-in tension in the resonator, and ρ is the mass density.

Supplementary Equation 14 shows that the resonant frequency of a doubly clamped resonator is a function of both its geometry and its intrinsic material properties. Two limits of the dynamic mechanical behavior happen when either one of the arguments under the square root become dominant over the other. For a thick resonator the first argument is dominant, the resonant frequency could be described as $f_0 = \frac{\pi t}{2L^2}\sqrt{E_Y/12\rho}$ and the resonator behaves in the so called "plate limit". The other extreme is the "membrane limit" where the resonator is very

thin and Supplementary Equation 14 is reduced to $f_0 = \frac{1}{2L}\sqrt{T_0/\rho_{2d}W}$, ρ_{2d} being the effective sheet density. Therefore, for resonators in the membrane limit, the resonant frequency is predominantly set by the tension rather than the bending rigidity ($\propto E_Y$) of the material.

In this work, in order to normalize for the different ribbon geometries, we use built-in strain in place of built-in tension. The built-in strain ε_0 is derived from the built-in tension T_0 using $T_0 = WtE_Y\varepsilon_0$ assuming E_Y = 270 GPa.[5]

Monolayer MoS_2 resonators present the ultimate limit of membrane thinness and are well in the membrane limit. Therefore, the presence of the built-in strain on the as-fabricated resonators accounts for the high resonant frequencies observed, even in the absence of any electrostatic DC force. In addition to the built-in strain, the gate voltage induced F_{es} results in additional electrostatic strain in the resonator. As a result of the additional strain, the resonant frequency shifts towards higher values. The shift in the resonant frequency could be described by the function Δ:

$$f_{res} - f_0(\varepsilon_0, \rho_{2d}) = \Delta(V_g, \varepsilon_0, \rho_{2d}) \quad (15)$$

which combines the effect of applied electrostatic strain, built-in strain and the mass density of the resonator. Chen et. al.[9] have developed a model that takes account of all these parameters and successfully describes the gate voltage dependence of the resonant frequency in atomically thin resonators. Using the same analytical model we plotted in Supplementary Figure 2 the predicted gate voltage induced resonance frequency tuning for various values of built-in strain and mass density, assuming a resonator with the same dimensions as R2. The model predicts that increasing the built-in strain (Supplementary Figure 2a) not only results in enhanced values of resonant frequency at V_g = 0, but also changes the curvature of from a convex to a concave shape. This prediction is also confirmed in experimental results showing a concave shape for higher built-in strain (R1) and convex curvature when the built-in strain is lower (R2). (Figure 3a and 3b from the main text). On the other hand, increasing the mass density (Supplementary Figure 2b) lowers the resonant frequency at V_g = 0 and reduces the sensitivity of the resonant frequency to the applied gate voltage, but, the curvature of the tuning curve does not change for different values of mass density.

Using the built-in strain and the mass density as the fitting parameters, we fit this model to the experimentally measured resonant frequency as a function of local gate voltage for 7 devices. The deduced values of mass density and built-in strain are shown in the Figure 3c in the main text. The limit to the tunability of the resonant frequency is set by the pull-in voltage at which F_{es} is large enough for the suspended resonator to snap in and collapse.

Supplementary Note 7: The equation of motion and the frequency response of a driven, damped Duffing resonator

Previous studies on the mechanical properties of MoS_2 have reported that the intrinsic strain-stress relation becomes nonlinear with increasing deformation.[5] However, in the case of our resonators, the nonlinear behavior appears at strain levels below the nonlinear regime in intrinsic stress-strain relation of MoS_2[5] and could be explained by the amplitude-dependent nonlinear effects in the equation of motion.

While for low driving force the resonators operate in the linear regime, increasing the driving force and the motional amplitude x result in appearance of nonlinear response. In this

regime, due to the increased x, the nonlinear restoring force (αx^3) and nonlinear damping ($\eta x^2\dot{x}$) should be taken into account and the equation of motion is described by:

$$\ddot{x}+\frac{k}{m}x+\left(\frac{\gamma}{m}+\frac{\eta}{m}x^2\right)\dot{x}+\frac{\alpha}{m}x^3+=\frac{F_0}{m}cos(\omega t) \tag{16}$$

known as the van der Pol-Duffing Equation[10] Here, x is the motional amplitude, k is the effective spring constant, m the resonator mass, γ is the linear damping coefficient, η is the nonlinear damping coefficient, α is the Duffing force coefficient, F_0 is the amplitude of the driving force, ω is the driving frequency and t is time.

Using secular perturbation theory, Lifshitz et. al. have calculated the solution of the van der Pol-Duffing equation in the limit of week linear damping (Q smaller than 1000).[11] The obtained expressions for the steady state motional amplitude x_0 and phase ϕ are:

$$x_0^2(\omega)=\frac{\left(\frac{F_0}{2m\omega_0^2}\right)^2}{\left(\frac{\omega-\omega_0}{\omega_0}-\frac{3}{8}\frac{\alpha}{m\omega_0^2}x_0^2(\omega)\right)^2+\left(\frac{1}{2Q}+\frac{1}{8}\frac{\eta}{m\omega_0}x_0^2(\omega)\right)^2} \tag{17}$$

and

$$\tan(\phi)=\frac{\frac{\gamma}{2}+\frac{\eta}{8}x_0^2(\omega)}{m(\omega-\omega_0)-\frac{3}{8}\frac{\alpha}{\omega_0}x_0^2(\omega)} \tag{18}$$

Inserting $\alpha=\eta=0$, reduces the above expressions to the response of a linear harmonic oscillator.

Supplementary Note 8: Effect of nonlinear restoring force and nonlinear damping on the frequency response

For high values of drive force and motional amplitude, nonlinear effects from Duffing force and nonlinear damping become considerable. In order to investigate the effect of nonlinear Duffing force, we consider the solutions to the Duffing resonator (Supplementary Equation 17) in the absence of nonlinear damping ($\eta=0$).

The Duffing force coefficient α could originate from two main sources; the nonlinearities arising from the presence of an external electrostatic force and the nonlinearities arising from geometric effects.

The external electrostatic drive force, applied between the resonator and the local gate electrode, results in the deviation of the resonator's equilibrium position from its rest position. The deviation from the rest position results in a cubic restoring force in the equation of motion (αx^3) which acts in the opposite direction of the linear elastic force (kx). Therefore, the resonator is softened and the resonant frequency reduces.[11] On the other hand, geometrical nonlinearities are expected to appear in doubly clamped atomically thin membranes where the transverse vibrations results in non-negligible elongation and stretching. In the case of geometric nonlinearities, Lifshitz et. al. have used a perturbative solution to the Euler-Bernoulli equation and derived the Duffing parameter, for the fundamental mode, which is described by

$$\alpha=\pi^4\rho_{2\mathrm{d}}Wf_0^2/L\varepsilon_0 \tag{19}$$

for a high-tension, thin, doubly clamped resonator.[11] Using the parameters we have derived for our representative device R2, we obtain $\alpha\sim 1.5\times10^{15}$ kg $\mathrm{m^{-2}s^{-2}}$. Furthermore, the positive Duffing coefficient leads to the stiffening and increase in the resonance frequency which is in

agreement with our observations indicating that, in the case of MoS_2 resonators reported in this work, the Duffing force originates from geometric nonlinearities.

Supplementary Figure 4a shows a schematic illustration of the resulting frequency response for increasing drive amplitude. The response to the small drive resembles the Lorentzian response of a driven harmonic oscillator with a symmetric peak at ω_0, and it becomes asymmetric as the drive increases. For a strong driving force, the Duffing equation could have two stable solutions referred to as the upper and the lower stable solutions.[11] As the drive frequency is swept from lower to higher values, the experimentally measured response follows the upper stable solution until it reaches the maximum point, and then falls to the lower stable solution which emerges as the abrupt change of the motional amplitude (instead of following the dashed line in Supplementary Figure 4a).

In practice, the condition for the existence of two stable solutions (bistability) is given by[11]:

$$\frac{\eta\omega_0}{\alpha} < \sqrt{3} \tag{20}$$

This allows extracting an upper limit for the nonlinear damping coefficient that would allow the bistability. Using the estimated value of $\alpha \sim 1.5 \times 10^{15}$ kg m^{-2}s^{-2}, we obtain $\eta <$ $\sim 4 \times 10^6$ kg m^{-2} s^{-1} for device R2, which is comparable with the values that have been reported for resonators based on carbon nanotubes and graphene.[12]

Bistability is also expected to give rise to hysteresis in the frequency response of Duffing resonators. However, the presence of thermal noise at room temperature, shrinks the size of the hysteresis loop and makes it undetectable in our measurements.[11]

The effect of nonlinear damping ($\frac{\eta}{m} x^2 \dot{x}$) appears as increased dissipation and broadening of the resonance peak. As long as the resonance bandwidth Δω is much smaller than the resonance frequency, the standard definition for the bandwidth is given by the ratio between the average energy loss rate and the average stored mechanical energy:

$$\Delta\omega = \frac{\langle {dE_\mathrm{m}}/{dt} \rangle}{\langle E_\mathrm{m} \rangle} = \frac{\omega_0}{Q} \tag{21}$$

where $\langle E_\mathrm{m} \rangle$ is the time-averaged stored mechanical energy over a few oscillation periods and $\langle {dE_\mathrm{m}}/{dt} \rangle$ is the average energy loss rate over the same period of time.

For a linear harmonic oscillator, the solution to the equation of motion results in $Q = \frac{m\omega_0}{\gamma}$. Therefore, the bandwidth could be written as $\Delta\omega = \frac{\omega_0}{Q} = \frac{\gamma}{m}$ which is independent of the amplitude of the mechanical vibrations, and thus, independent of the driving force. In this regime, the frequency response of the mixing current shows a symmetric lineshape, and, the peak frequency is the same for the mixing current as the stored mechanical energy. Figures 2a and Supplementary Figure 4b show the frequency response of the mixing current for devices R1 and R2, respectively, measured at low drive amplitude where the resonator is in the linear regime. Hence, we can fit the peaks with a Lorentzian fit (dashed red lines in Figures 2a and the Supplementary Figure 4b). We extract values of Q = 225 and $\frac{\Delta\omega}{2\pi}$ = 500 kHz for R1 Q = 75 and $\frac{\Delta\omega}{2\pi}$ = 1.6 MHz for R2, and estimated the value of γ using $\gamma = m\Delta\omega$ resulting in $\gamma_{R1} = 1.4 \times 10^{-11}$ kg s^{-1} and $\gamma_{R2} = 1.2 \times 10^{-11}$ kg s^{-1}.

The resonators enter the nonlinear regime when the driving force is increased. In the nonlinear regime, the effective damping is a superposition of the linear and nonlinear damping expressed as $\gamma_{eff} = \gamma + \frac{1}{4}\eta x_0^2$.[11] It is possible to estimate the gate voltage at which the

influence of nonlinear damping becomes considerable by looking at the responsivity R defined as:

$$R \triangleq I_{\mathrm{peak}}/F_0 \propto I_{\mathrm{peak}}/V_{\mathrm{g}} \tag{22}$$

Here I_{peak} is the maximum of mixing current. The resulting responsivity is plotted as a function of V_{g} in Figure 4b in the main text with a peak observed around V_{g} = 4 V which indicates the gate voltage at which the contribution from nonlinear damping to the effective dissipation become considerable.

At sufficiently high drive amplitudes, where the nonlinear damping becomes appreciable, the resonance width and the quality factor show strong dependence on the drive amplitude. Supplementary Figure 4c shows the resonance width as a function of the local gate voltage for R2. We used a model derived by Eichler et. al. to fit the experimental results and extracted the quality factor (right hand axis on Supplementary Figure 4c) for the asymmetric peaks, assuming a regime where $\gamma_{eff} \approx \frac{1}{4}\eta x_0^2$ [12] which holds true for $x_0 > 2.75$ nm. The observed nonlinear damping is consistent with observations in carbon nanotubes and graphene resonators[12] and confirms that in contrast to larger resonators (from meter-scale to tens of nanometer-scale), for resonators with atomic-scale transverse dimensions, damping becomes highly dependent on the amplitude of mechanical vibrations. Further investigation of the nonlinear behavior in MoS_2 resonators allows eliminating it when it is unwanted and efficiently using it when it is desirable. Additionally, the observed nonlinear behavior sheds light on the potential of MoS_2 resonators for studying nonlinear mode coupling between various vibrational modes, internal resonances and energy transfer from one mode to another.[8]

Supplementary Note 9: Dynamic range and mass resolution

Presence of nonlinear behavior in MoS_2 NEMS resonators makes it relevant to extract the linear dynamic range of these resonators. The critical mechanical amplitude at which the nonlinear behavior sets in, can be estimated from the equation[8,13]:

$$a_{\mathrm{c}} = 1.5\sqrt{\frac{1}{Q}\left(\frac{t^2}{3} + \frac{TL^2}{\pi^2 E_{\mathrm{Y}} W t}\right)} \tag{23}$$

here t is thickness, Q is the quality factor in the linear regime and T the tension experienced by the resonator at the onset of the nonlinearity (extracted from the resonant frequency for the corresponding V_{g}).

The 1 dB dynamic range (DR) is given by[13]:

$$DR(dB) = 20\log\left(\frac{0.745 a_{\mathrm{c}}}{\sqrt{2S\Delta f}}\right) \tag{24}$$

with Δf being the measurement bandwidth and S the spectral density of the thermomechanical noise at resonant frequency given by[14,15]:

$$S = 4k_{\mathrm{B}}TQ/M_{\mathrm{eff}}\omega_0^3 \tag{25}$$

here k_{B} is the Boltzmann constant, T is the temperature and M_{eff} is the effective mass for the fundamental mode of a doubly clamped resonator given by $M_{\mathrm{eff}} = 0.735\ LW\rho_{2\mathrm{d}}$.

Moreover, we can use the obtained dynamic range to deduce the mass resolution of the resonator[15]:

$$\delta m \approx \frac{2M_{\text{eff}}}{Q} \times 10^{-DR(\text{dB})/20} \tag{26}$$

Supplementary Table 1 shows the values extracted for devices R1 and R2 from the main text:

	a_c (nm)	DR (dB)	δm (attogram)
R1	~2	~75	~5×10^{-3}
R2	~1.8	~73.5	~4.5×10^{-3}

Supplementary Table 1.

Comparing the values derived for R1 and R2 reveals that the higher values of built-in strain result in enhanced critical amplitude and improve the dynamic range. This observation is consistent with the theoretical predictions[16] and suggests that strain could be used to reduce the nonlinear behavior of NEMS resonators which is an inevitable by-product of miniaturization.

Supplementary Note 10: Effect of input RF power on the frequency response

As a result of increasing the input RF power and amplitude dependent tension, the frequency at which the maximum of the mixing current occurs (ω_{p}) deviates from the frequency of linear harmonic oscillator (ω_0). Lifshitz et. al. use the secular perturbation theory, to show that this deviation is described by

$$\omega_{\text{p}} - \omega_0 \sim \frac{3\alpha x_{\text{p}}^2}{8\omega_0 m} \tag{27}$$

with x_{p} the maximum of the mechanical motion amplitude, α the Duffing force coefficient and m the resonator mass.[11]

Combining Supplementary Equations 13, 17 and 27, the dependence of $\omega_{\text{p}} - \omega_0$ on input RF power $P_{\text{in}}^{\text{RF}}$ is derived as:

$$\left(\omega_{\text{p}} - \omega_0\right) \propto x_{\text{p}}^2 \propto F_0^{\frac{2}{3}} \propto V_{\text{c}}^{\frac{2}{3}} \propto \left(P_{\text{in}}^{\text{RF}}\right)^{\frac{1}{3}} \tag{28}$$

The black line in Figure 4d in the main text is a fit to this power dependence.

Additionally, the peak value of the mixing current increases by increasing the input RF power. According to Supplementary Equation 1 the dependence of the mixing current on the input RF power is described as:

$$I_{\text{mix}}^{\text{FM}} \propto V_{\text{c}}\, \alpha \left(P_{\text{in}}^{\text{RF}}\right)^{\frac{1}{2}} \tag{29}$$

Supplemententry Figure 5 shows the experimentally measured peak current as a function of the input RF power (red markers) and the fit to $\left(P_{\text{in}}^{\text{RF}}\right)^{\frac{1}{2}}$ (black line).

Supplementary Note 11: Effect of the suspended contacts

Releasing the MoS_2 channel by undercutting the oxide layer in a wet etching step offers the advantage that the distance between the suspended MoS_2 and the substrate is uniform under all the channel area. This is due to the fact that in the lateral interface between MoS_2 and the oxide, the acid diffuses rapidly and Al_2O_3 is etched at the same rate in all the interface area.[17] The MoS_2-Al_2O_3 interface area includes the region of the MoS_2 flake which is contacted by metal electrodes, therefore, the metal electrodes in the clamping region are also suspended.

Due to the finite rigidity of the metal contacts, vibrations of the MoS_2 membrane are extended in the suspended region of the contacts. The resonance of the undercut metal electrodes result in the appearance of additional peaks in the frequency response of the mixing current. These peaks are easy to identify since they show lower quality factors and their resonant frequency is not sensitive to the gate voltage. Supplementary Figure 7a shows the frequency response for a 1.1 μm long and 92 nm wide MoS_2 resonator at V_g = 2.5 V. The prominent peak observed near 123 MHz corresponds to the mechanical resonance of the MoS_2 channel and has a width of ∼0.6 MHz. The broader peak observed at 129 MHz is attributed to the mechanical vibrations of the suspended portion of the electrodes and shows a width ∼5 MHz. Supplementary Figure 7b shows the mixing current mapped as a function of the local gate voltage and the driving frequency. The dashed lines serve as guides to the eye. The difference between the tunability of the MoS_2 resonance and the electrode's resonance with V_g further confirms the origins of the two peaks. The MoS_2 resonators operate in the membrane limit where the resonant frequency is tunable via the gate voltage induced static strain, while the thick metal electrodes behave in the plate limit where the resonant frequency is predominantly defined by the bending rigidity and is independent of the applied strain. Once it shifts close to the resonant frequency of the contacts, the resonant frequency of the MoS_2 membrane could not be detected in the background. By further increasing the local gate voltage, the resonant frequency of the MoS_2 membrane surpasses the resonant frequency of the suspended contacts and could be detected again (Supplementary Figure 7c). Supplementary Figure 7d shows the frequency response of the mixing current for V_g = 10.5 V. The MoS_2 resonant peak is then occurring at frequencies above the resonant frequency of the suspended contacts. Furthermore, the higher value of drive amplitude in Supplementary Figure 7d results in higher current levels and appearance of nonlinearity, in comparison to Supplementary Figure 7a.

Behaving in the plate limit due to their relatively large thickness (typically around 80 nm) and bending rigidity, the resonant frequency of the suspended gold contacts is proportional to $(1/L_{\mathrm{metal}})^2$, L_{metal} being the length of the suspended part of the electrode (Supplementary Note 6). The suspended part of the electrode is mainly in the clamping region meaning that L_{metal} ~ width of the MoS_2 membrane. Due to the narrow width of the ribbons measured in our experiment, the resonance from the suspended metal contacts rarely appeared in the frequency range where we observed the electromechanical resonance from the MoS_2 NEMS resonators. In the two cases where we have observed such peaks, the peaks where at 129 MHz and 114 MHz for devices of width 92 nm and 98.5 nm respectively, which is consistent with the resonant frequency being proportional to $(1/L_{\mathrm{metal}})^2$.

SUPPLEMENTARY REFERENCES

1. Dumcenco, D. *et al.* Large-Area Epitaxial Monolayer MoS_2. *ACS Nano* **9,** 4611–4620 (2015).
2. Gouttenoire, V. *et al.* Digital and FM demodulation of a doubly clamped single-walled carbon-nanotube oscillator: towards a nanotube cell phone. *Small* **6,** 1060–1065 (2010).
3. Manzeli, S., Allain, A., Ghadimi, A. & Kis, A. Piezoresistivity and Strain-induced Band Gap Tuning in Atomically Thin MoS_2. *Nano Lett.* **15,** 5330–5335 (2015).
4. Benameur, M. M. *et al.* Electromechanical oscillations in bilayer graphene. *Nat. Commun.* **6,** (2015).
5. Bertolazzi, S., Brivio, J. & Kis, A. Stretching and Breaking of Ultrathin MoS_2. *ACS Nano* **5,** 9703–9709 (2011).
6. Birey, H. Thickness dependence of the dielectric constant and resistance of Al_2O_3 films. *J. Appl. Phys.* **48,** 5209–5212 (1977).
7. Landau, L. D. & Lifshitz, E. M. *Theory of Elasticity - 3rd Edition*. (Butterworth-Heinemann, 1986).
8. Nayfeh, Ali. H. & Mook, Dean. T. *Nonlinear Oscillations, Physics and Applied Mathematics*. (John Wiley, New York, 1979).
9. Chen, C. *et al.* Performance of monolayer graphene nanomechanical resonators with electrical readout. *Nat. Nanotechnol.* **4,** 861–867 (2009).
10. Zaitsev, S., Shtempluck, O., Buks, E. & Gottlieb, O. Nonlinear damping in a micromechanical oscillator. *Nonlinear Dyn.* **67,** 859–883 (2012).
11. Lifshitz, R. & Cross, M. C. *Reviews of Nonlinear Dynamics and Complexity*. **1,** (Wiley-VCH, 2008).
12. Eichler, A. *et al.* Nonlinear damping in mechanical resonators made from carbon nanotubes and graphene. *Nat. Nanotechnol.* **6,** 339–342 (2011).
13. Postma, H. W. Ch., Kozinsky, I., Husain, A. & Roukes, M. L. Dynamic range of nanotube- and nanowire-based electromechanical systems. *Appl. Phys. Lett.* **86,** 223105 (2005).
14. Robins, W. P. *Phase Noise in Signal Sources: Theory and Applications*. (Peter Peregrinus Limited, 1982).
15. Ekinci, K. L., Yang, Y. T. & Roukes, M. L. Ultimate limits to inertial mass sensing based upon nanoelectromechanical systems. *J. Appl. Phys.* **95,** 2682–2689 (2004).
16. Younis, M. I. & Nayfeh, A. H. A Study of the Nonlinear Response of a Resonant Microbeam to an Electric Actuation. *Nonlinear Dyn.* **31,** 91–117 (2003).
17. Bolotin, K. I. *et al.* Ultrahigh electron mobility in suspended graphene. *Solid State Commun.* **146,** 351–355 (2008).
